# The effect of short music videos on needs satisfaction and separation anxiety of children's family members during COVID-19: The example of TikTok

**DOI:** 10.3389/fped.2022.990752

**Published:** 2022-09-07

**Authors:** Ya-Li Huang, Yu-Kun Chen, Shi-Hao Lin, Hua Cao, Qiang Chen

**Affiliations:** ^1^Department of Cardiac Surgery, Fujian Branch of Shanghai Children's Medical Center, Fuzhou, China; ^2^Department of Cardiac Surgery, Fujian Children's Hospital, Fuzhou, China; ^3^College of Clinical Medicine for Obstetrics and Gynecology and Pediatrics, Fujian Medical University, Fuzhou, China; ^4^Fujian Key Laboratory of Women and Children's Critical Diseases Research, Fujian Maternity and Child Health Hospital, Fuzhou, China

**Keywords:** COVID-19, music short video, separation anxiety, needs satisfaction, ICU

## Abstract

**Objective:**

To investigate the effect of short music videos on needs satisfaction and separation anxiety of the family members of children with congenital heart disease (CHD) in the cardiac intensive care unit (CICU) during the COVID-19 pandemic.

**Methods:**

Eighty-seven children's family members were divided into the study group and the control group between February 2020 and March 2021. During the COVID-19 pandemic, the participants in the control group were visited by telephone, while the participants in the study group used the TikTok short music video application and WeChat as communication tools. After the intervention, the critical care family needs inventory (CCFNI) and separation anxiety scale (SAS) for all participants were recorded and analyzed.

**Results:**

There were no statistically significant differences in general characteristics or preintervention data between the two groups. However, the two dimensions of the support scale and information scale of the CCFNI in the study group were significantly different from those in the control group after the intervention (*P* = 0.008, and *P* = 0.021, respectively). There were significant differences in the three dimensions of the SAS between the two groups (*P* = 0.004, *P* = 0.007, and *P* = 0.041, respectively).

**Conclusion:**

The visiting system of the ICU changed during the COVID-19 pandemic, and the use of the TikTok short music video application and WeChat was conducive to optimizing the CICU ward visiting process, reducing the separation anxiety of the family members of children in the CICU, and improving their needs satisfaction.

## Introduction

The cardiac intensive care unit (CICU) is a special place for rescuing critical children with congenital heart disease (CHD), so the visitation process must be strictly managed. After cardiac surgery, children are transferred to the CICU. These children not only have to bear the pain of the surgical incision but also need to adapt to a new environment in a short time. This situation easily induces and aggravates the separation anxiety between children and their families. It is well-known that the ICU is a closed ward, and visits are limited, which can cause separation anxiety in children's parents. Being with children was the most urgent need of children's parents (1, 2). During the COVID-19 pandemic, the ICU visiting system directly affected the prevention and control of hospitals (3). The ICU ward implemented no escort system, refused visits from family members, and strictly implemented infection prevention and control measures, all to reduce the risk of infection to the maximum. The family members may be under great anxiety because they cannot see their children during this critical time.

A systematic review suggests that flexible ICU visiting hours were associated with lower severity of anxiety symptoms of the family members and improved their needs satisfaction (4). However, the lack of an escort system will exacerbate the anxiety of the families, which not only affects their health but also the subsequent treatment of their children. Studies showed that 10–42% of the family members of patients in the ICUs of American hospitals had anxiety symptoms (5). A higher prevalence was found in another study from 78 French ICUs, in which 73% of family members had anxiety symptoms (6, 7). Lopez et al. (8) showed that the family members of children with CHD were more likely to have symptoms of depression, anxiety, and despair than those of healthy children. Landolt et al. (9) found that the overall mental health of the family members of children with CHD decreased, and there were often adverse emotions such as insomnia, anxiety, and depression. For family members of children who had just completed the operation and stayed in the ICU, in particular, this was a negative life event and a strong psychological stress for them. The worry about children's physical condition, the change of role, coupled with financial burdens and other factors were likely to make the family members experience anxiety (10, 11). Most researchers pay attention to the treatment and prevention of complications in children with CHD. However, less attention has been given to the psychological problems of their family members.

Tiktok is a music creative short video social software developed by ByteDance (Beijing, China). TikTok and WeChat are popular social media platforms used in China. Studies have shown that TikTok is a simple, effective, noninvasive, and inexpensive method to reduce preoperative anxiety. TikTok could reduce patients' blood pressure and heart rate to a certain extent (12). Moreover, TikTok has been increasingly widely used in the dissemination and promotion of health-related information in the medical field (13, 14). This study aimed to assess the effect of short music videos made by TikTok and delivered *via* WeChat, which was used as a new psychological intervention to relieve separation anxiety and improve the satisfaction of the needs of the family members of children with CHD living in the CICU.

## Methods

### Ethical approval

A before-and-after controlled clinical study was conducted in a provincial children's hospital in China. The study was approved by the hospital ethics committee. The questionnaire in this study was anonymous, and the participants gave informed consent before participating in the survey.

### Sample size and distribution

For the sample size calculation, the a value was set at 0.05 with a power of 0.90. Based on the results of the preliminary study and using a 10% loss to follow-up rate, the required number of participants in each group was calculated to be 45, and the total sample size was 90. The participants were selected by a successive sampling method: 1–45 were defined as the control group, and 46–90 were defined as the study group.

### Participant recruitment

Our hospital provided the setting from February 2020 to March 2021 for this study. A sample of 90 participants was recruited. The inclusion criteria for participants were as follows: (1) patients aged 0–7 years; (2) patients with simple CHD (atrial septal defect, ventricular septal defect, atrial septal defect with ventricular septal defect, etc.) diagnosed by transthoracic echocardiography; (3) patients who had received surgical correction by sternotomy or thoracotomy; (4) patients admitted to the CICU ward for the first time; (5) family members were the primary caregivers of children and were responsible for medical decision-making; (6) family members had certain reading, writing, and expression skills; (7) family members were proficient in using a smartphone; and (8) they followed the ethical standards and signed the informed consent. Exclusion criteria were as follows: (1) the patients had complications with other system diseases; (2) the family members had complications with mental diseases or emotional disorders such as anxiety neurosis, phobia, or personality disorder; and (3) the family members were unable to complete the study.

### Measurements

The needs of the family members were measured by using the critical care family needs inventory (CCFNI) (15, 16). There are 45 items in five dimensions: support scale (SS) (15 items), comfort scale (CS) (6 items), information scale (IS) (8 items), proximity scale (PS) (9 items), and assurance scale (AS) (7 items), with 45 items in total. The 4-point Likert scale is adopted for each demand: 1 for unimportant; 2 for important; 3 for more important; 4 for very important. The higher the score, the more needs are required. Cronbach's α coefficient of the scale is 0.88–0.98, and Cronbach's α coefficient of the 5 factors is 0.61–0.88, which has good reliability and validity for family needs (17).

Separation anxiety scales (SAS) have been proven by Aurora to be effective, reliable, and clinically practical in assessing family separation anxiety (18). A presurvey was conducted on the families of 30 children to study whether each item was in line with the expression habits and easy to understand. Cronbach's α coefficient of the scale is 0.89. The scale includes 35 questions in three dimensions: family members' perception of separation, family members' attitudes toward separation from the children, and family members' worry about separation caused by work. The scale adopts a 5-point scale, ranging from 1 to 5 points. The higher the score, the more serious the anxiety.

### Intervention

The family members of the children in the control group were contacted by medical staff by telephone. Visit communication was carried out at the specified time (15:00–15:30 every day). The family members in the study group received the short music video of children in the CICU, which was recorded *via* TikTok and delivered *via* WeChat each day. The methods in the study group were as follows. 1. Preliminary preparation. A special WeChat account should be set up by the family members. After that, the researchers were responsible for establishing a WeChat communication group with mobile phones. Members of the WeChat group include children's families, researchers, and doctors. 2. Implementation of the TikTok short music video application. The implementation was 3 times a day, at 06:00, 14:00, and 22:00. The video was taken by the researchers using TikTok. The shooting time was 15 s, the content of the video mainly reflected the current state of the patient, and the head area of the video screen was the best 2/3 of the full screen. The background music of the video should be relaxed and cheerful rather than sad and depressed. After the short video TikTok was recorded, and the physician introduced the vital signs of the patients. After the patients were transferred to the ward, the researchers deleted the video. This process was carried out by a dedicated researcher on our team to minimize the bias generated during this process.

### Data collection

All participants who met the inclusion criteria were enrolled in this study. The research team explained the purpose and significance of the research to the participants and obtained their understanding and support. Then, the research team further explained the precautions and methods for filling out the questionnaire according to the unified guidance, which should be completed by the participants independently. The general information questionnaire, CCFNI, and SAS were collected on the day when the patients were removed from the CICU. The general information questionnaire included the age, gender, education level, marital status, income, payment method of medical expenses, and religious beliefs of each child's family members. The child's age, gender, whether the child was an only child, and type of disease were also collected. The data collection was jointly completed by the main researchers trained in this study.

### Statistical analysis

Data were analyzed using SPSS (version 19.0 for Windows, IBM Corporation). Quantitative variables were expressed as the means ± standard deviations, and their distributions were checked for normality. The Mann-Whitney *U*-test was used to compare the quantitative variables that did not have a normal distribution. The Student's *t*-test was used for quantitative variables that followed a normal distribution. The χ^2^ test or Fisher's test was used to categorize variables. A *p*-value < 0.05 was considered statistically significant.

## Results

A total of 90 participants were included in the study. In the control group, one participant decided to withdraw from the study due to financial reasons, and two withdrew for personal reasons. The results of 42 participants in the control group and 45 in the study group were analyzed. No significant differences were found in age, gender, education level, marital status, family monthly income, payment method of medical expenses, or religious beliefs of the family members between the two groups. There was also no significant difference in age, sex, whether the child was an only child, or the type of CHD between the two groups (*P* > 0.05) (Table 1).

**Table 1:** Demographic and baseline characteristics of participants.

**Variable**	**Control group (*n* = 42), *n* (%)**	**Study group (*n* = 45), *n* (%)**	***P*-value**
**Children**			
Age, year	2.69 ± 2.22	2.45 ± 2.01	0.592
**Gender**			
Male	23 (54.8)	27 (60.0)	0.621
Female	19 (45.2)	18 (40.0)	
**Only child**			
Yes	16 (38.1)	20 (44.4)	0.548
No	26 (61.9)	25 (55.6)	
**Types of CHD**			
ASD	6 (14.3)	8 (17.7)	0.830
VSD	31 (73.8)	30 (66.7)	
Others	5 (11.9)	7 (15.6)	
**Family members**			
Age, year	26.42 ± 1.55	26.53 ± 1.57	0.739
**Gender**			
Male	6 (14.3)	5 (11.1)	0.753
Female	36 (85.7)	40 (88.9)	
**Marital status**			
Married	39 (92.9)	41 (91.1)	0.539
Others	3 (7.1)	4 (8.9)	
**Religious belief**			
Yes	11 (26.2)	12 (26.7)	0.960
No	31 (73.8)	33 (73.3)	
**Education level**			
Junior high school	5 (11.9)	6 (13.3)	0.773
Senior high school	17 (40.5)	21 (46.7)	
Universities and higher	20 (47.6)	18 (40.0)	
**Family monthly income (yuan)**			
<5000	13 (31.0)	9 (20.0)	0.494
5000~10000	22 (52.4)	28 (62.2)	
>10000	7 (16.7)	8 (17.8)	
**Payment of medical cost**			
Self-financed	5 (11.9)	6 (13.3)	0.961
Medical insurance	31 (73.8)	32 (71.1)	
Others	6 (14.3)	7 (15.6)	

Before the intervention, there was no significant difference in CCFNI or SAS scores between the two groups (*P* > 0.05). After the intervention, the CCFNI scores were higher in the study group than in the control group at SS and IS, and the difference was statistically significant (*P* = 0.008, and *P* = 0.021, respectively) (Table 2). The SAS scores in the study group were significantly lower than those in the control group (*P* = 0.004, *P* = 0. 07, and *P* = 0.041, respectively) (Table 3).

**Table 2:** Comparison of scores of CCFNI before and after the intervention between the two groups.

**Variable**	**Control group (*n* = 42)**	**Study group (*n* = 45)**	***P*-value**
**Pre-test**
SS	2.74 ± 0.62	2.77 ± 0.52	0.748
CS	2.05 ± 0.66	2.13 ± 0.59	0.524
IS	2.74 ± 0.73	2.78 ± 0.70	0.798
PS	3.00 ± 0.69	3.0 ± 0.72	0.663
AS	2.93 ± 0.64	2.73 ± 0.69	0.175
**Post-test**			
SS	2.76 ± 0.61	3.13 ± 0.66	0.008
CS	2.21 ± 0.65	2.24 ± 0.57	0.818
IS	2.83 ± 0.76	3.20 ± 0.69	0.021
PS	3.12 ± 0.71	3.16 ± 0.71	0.810
AS	3.02 ± 0.64	3.15 ± 0.88	0.430

**Table 3:** Comparison of scores of SAS before and after the intervention between the two groups.

**Variable**	**Control group (*n* = 42)**	**Study group (*n* = 45)**	***P*-value**
**Pre-test**			
Perception of separation	2.95 ± 0.73	3.09 ± 0.70	0.377
Attitude of separation	3.47 ± 0.77	3.56 ± 0.69	0.615
Concerns about separation caused by work	3.30 ± 0.72	3.33 ± 0.64	0.870
**Post-test**			
Perception of separation	2.81 ± 0.64	2.37 ± 0.72	0.004
Attitude of separation	3.38 ± 0.76	2.89 ± 0.88	0.007
Concerns about separation caused by work	3.12 ± 0.67	2.76 ± 0.93	0.041

## Discussion

A study from Greece showed that the incidence of anxiety and depression in the family members of ICU patients was as high as 97% (19). When the family members of ICU patients were faced with uncertain factors such as serious illness and life danger at any time, they often had anxiety, depression, sleep disorders, acute stress disorder, and posttraumatic stress disorder, which would seriously affect the physical and mental health of the family members (20). Families of children in the CICU must face complex medical procedures, the potentially fatal risk of cardiac surgery, and the risk of postoperative complications. The short-term separation from children, the plight of children in the CICU, postoperative pain and further treatment, and the increasing pressure on children after admission also make their families prone to anxiety. As the main component of the ICU patient support system, family members play a vital role in children's physiological and psychological rehabilitation (21).

In the context of a new coronavirus pneumonia pandemic prevention and control, the implementation of the protocol that restricts visiting does not meet the needs of the family members visiting children daily, thereby increasing their anxiety. Therefore, during the coronavirus pneumonia pandemic situation, we should not only consider the treatment and nursing needs of children but also consider the needs of the family members of children (22). At present, the visiting-related modes and systems have not been unified at home and abroad, and there are still certain differences in their specific implementation. The implementation effect varies from country to country. Most hospitals in Canada do not allow visits, and ICUs provide virtual visits when prohibiting visits (23). Many hospitals in the United States use alternative means of communication through frequent telephone and virtual visits to ensure clinical updates and maintain family contacts (24). At present, the visiting management of ICUs in China needs to seek a visiting system combining management and humanity according to Chinese national conditions. Some hospitals have begun to design ICU mobile app platforms. The overall design of the app needs to be completed by professional development teams, hospital information centers, coordinated software engineers, and ICU medical staff. In the transitional stage of accelerating the reform of the ICU visiting system, medical staff should take more active measures to promote the connection of information inside and outside the ICU to meet the psychological needs of family members. The study found that the communication time between the medical staff and the family members was generally <10 min. The only way to reduce the anxiety of ICU patients' families was to increase the contact between the ICU medical staff and the family members and provide them with sufficient relevant information (25).

TikTok is a short video software with a headline developed in September 2016. It combines visual and auditory technology. It has the advantage of being a short, flat, and fast communication, and it is simple and easy to operate. TikTok has great potential to convey important public health information to different populations during the COVID-19 pandemic (26, 27).

The results of this study showed that the SS and IS of CCFNI in the study group were significantly higher than those in the control group, indicating that the use of TikTok short music videos were able to improve the degree of satisfaction of family members for medical needs and achieve the purpose of offering humanistic care. When children were admitted to the CICU after cardiac surgery, family members were prone to anxiety and had other negative emotions, which was consistent with the research results of Uhm and his team about the anxiety of mothers of children with CHD operations (28). Our results also showed that the perception of separation, the attitude toward separation, and concerns about separation in the study group were significantly lower than those in the control group, indicating that short TikTok music videos could reduce the anxiety of family members. On the one hand, this might be because the short music video shared the daily situation of the children, which reduced the anxiety of the family members due to the prohibition on visiting. On the other hand, through the repeated playback function of TikTok, listening to the music and watching the video established the family members' confidence in overcoming diseases and letting them to greatly relieve their anxiety. Therefore, the TikTok short music video was informational, effectively avoided the phenomenon of family members gathering and was conducive to strictly implementing the coronavirus prevention and control technology guide requirements, minimizing the risk of an infection outbreak and solving the problem of visiting during the pandemic. In short, TikTok had become a new means for visiting in hospitals during these special times. At the same time, the application of TikTok music and short videos reflected the awareness of medical staff of the importance of providing active services through new technologies to achieve a mutual win-win between the hospital and the patients' families and to meet the visiting needs during the pandemic period.

## Limitations

This study had the following limitations. First, the participants of this study were limited to the family members of children with CHD after surgery, and there was no comparison between the psychological state of our participants and the family members of their children with other diseases or healthy children. Second, the intervention time was short, and the evaluation results of the TikTok short music videos were few. Further study should be completed to evaluate the improvement effect of the family members' cognition and health information. Third, the effects of music and nonmusic live videos and recorded videos using TikTok and Live Video were not compared. Because of the setting of video production permission, the impact of video capacity on the results was not studied. The study was also limited by these conditions and the lack of a large-sample, multicenter clinical trial design. Therefore, large-sample, multicenter controlled trials are warranted.

## Conclusion

During the COVID-19 pandemic, the use of short music videos by TikTok was conducive to optimizing the CICU visiting process, reducing the separation anxiety of the family members of children in the CICU, and improving their needs satisfaction.

## Data availability statement

The original contributions presented in the study are included in the article/supplementary material, further inquiries can be directed to the corresponding author.

## Ethics statement

The studies involving human participants were reviewed and approved by the Ethics Committee of Fujian Children's Hospital. Written informed consent to participate in this study was provided by the participants' legal guardian/next of kin.

## Author contributions

Y-LH and QC designed the study, performed the statistical analysis, participated in the operation, and drafted the manuscript. Y-KC, S-HL, and HC collected the clinical data. All authors read and approved the final manuscript.

## Conflict of interest

The authors declare that the research was conducted in the absence of any commercial or financial relationships that could be construed as a potential conflict of interest.

## Publisher's note

All claims expressed in this article are solely those of the authors and do not necessarily represent those of their affiliated organizations, or those of the publisher, the editors and the reviewers. Any product that may be evaluated in this article, or claim that may be made by its manufacturer, is not guaranteed or endorsed by the publisher.

## References

[1] Engström Å Dicksson E Contreras P. The desire of parents to be involved and present. Nurs Crit Care. (2015) 20:322–30. 10.1111/nicc.12103 25270994

[2] Kasper JW Nyamathi AM. Parents of children in the pediatric intensive care unit: what are their needs? Heart Lung. (1988) 17:574–81. 3417468

[3] Government of China. Notice on Issuing Technical Guidelines for Prevention and Control of Coronavirus Infection in Medical Institutions. Available online at: http://www.nhc.gov.cn/yzygj/s7659/202001/b91fdab7c304431eb082d67847d27e14.shtml (accessed January 2020)

[4] Nassar AP Besen BAMP Robinson CC Falavigna M Teixeira C Rosa RG. Flexible vs. restrictive visiting policies in ICUs: a systematic review and meta-analysis. Crit Care Med. (2018) 46:1175–80. 10.1097/CCM.0000000000003155 29642108

[5] Siegel MD Hayes E Vanderwerker LC Loseth DB Prigerson HG. Psychiatric illness in the next of kin of patients who die in the intensive care unit. Crit Care Med. (2008) 36:1722–8. 10.1097/CCM.0b013e318174da72 18520637

[6] Trevick SA Lord AS. Post-traumatic stress disorder and complicated grief are common in caregivers of neuro-ICU patients. Neurocrit Care. (2017) 26:436–43. 10.1007/s12028-016-0372-5 28054288

[7] Kiecolt-Glaser JK McGuire L Robles TF Glaser R. Emotions, morbidity, and mortality: new perspectives from psychoneuroimmunology. Annu Rev Psychol. (2002) 53:83–107. 10.1146/annurev.psych.53.100901.135217 11752480

[8] López R Frangini P Ramírez M Valenzuela PM Terrazas C Pérez CA. Well-being and agency in parents of children with congenital heart disease: a survey in chile. World J Pediatr Congenit Heart Surg. (2016) 7:139–45. 10.1177/2150135115623284 26957395

[9] Landolt MA Ystrom E Stene-Larsen K Holmstrøm H Vollrath ME. Exploring causal pathways of child behavior and maternal mental health in families with a child with congenital heart disease: a longitudinal study. Psychol Med. (2014) 44:3421–33. 10.1017/S0033291713002894 24286537

[10] Grov EK Dahl AA Moum T Fosså SD. Anxiety, depression, and quality of life in caregivers of patients with cancer in late palliative phase. Ann Oncol. (2005) 16:1185–91. 10.1093/annonc/mdi210 15849218

[11] Miles MS Carter MC Spicher C Hassanein RS. Maternal and paternal stress reactions when a child is hospitalized in a pediatric intensive care unit. Issues Compr Pediatr Nurs. (1984) 7:333–42. 6571117 10.3109/01460868409009770

[12] Gu S Ping J Xu M Zhou Y. TikTok browsing for anxiety relief in the preoperative period: a randomized clinical trial. Complement Ther Med. (2021) 60:102749. 10.1016/j.ctim.2021.102749 34118388

[13] Kong W Song S Zhao YC Zhu Q Sha L. TikTok as a health information source: assessment of the quality of information in diabetes-related videos. J Med Internet Res. (2021) 23:e30409. 10.2196/30409 34468327 PMC8444042

[14] Kriegel ER Lazarevic B Athanasian CE Milanaik RL. TikTok, Tide Pods and Tiger King: health implications of trends taking over pediatric populations. Curr Opin Pediatr. (2021) 33:170–7. 10.1097/MOP.0000000000000989 33337608

[15] Leske JS. Needs of relatives of critically ill patients: a follow-up. Heart Lung. (1986) 15:189–93. 3633247

[16] Chien WT Ip WY Lee IY. Psychometric properties of a Chinese version of the critical care family needs inventory. Res Nurs Health. (2005) 28:474–87. 10.1002/nur.20103 16287056

[17] Leske JS. Comparison of family stresses, strengths, and outcomes after trauma and surgery. AACN Clin Issues. (2003) 14:33–41. 10.1097/00044067-200302000-00005 12574701

[18] Orenes A García-Fernández JM Méndez X. Separation anxiety assessment scale-parent version: Spanish validation (SAAS-P: Spanish validation). Child Psychiatry Hum Dev. (2019) 50:826–34. 10.1007/s10578-019-00885-6 30903436

[19] Iwata M Han S Hays R Doorenbos AZ. Predictors of depression and anxiety in family members 3 months after child's admission to a pediatric ICU. Am J Hosp Palliat Care. (2019) 36:841–50. 10.1177/1049909119859517 31256606

[20] Płaszewska-Zywko L Gazda D. Emotional reactions and needs of family members of ICU patients. Anaesthesiol Intensive Ther. (2012) 44:145–9. 23110291

[21] Schnell JL Johaningsmeir S Bartelt T Bergman DA. Partnering with parents of children with medical complexity: a framework for engaging families for practice improvement. Pediatr Ann. (2020) 49:e467–72. 10.3928/19382359-20201012-01 33170294

[22] Choi J Donahoe MP Hoffman LA. Psychological and physical health in family caregivers of intensive care unit survivors: current knowledge and future research strategies. J Korean Acad Nurs. (2016) 46:159–67. 10.4040/jkan.2016.46.2.159 27182013

[23] Fiest KM Krewulak KD Hiploylee C Bagshaw SM Burns KEA Cook DJ. An environmental scan of visitation policies in Canadian intensive care units during the first wave of the COVID-19 pandemic. Can J Anaesth. (2021) 68:1474–84. 10.1007/s12630-021-02049-4 34195922 PMC8244673

[24] Sasangohar F Dhala A Zheng F Ahmadi N Kash B Masud F. Use of telecritical care for family visitation to ICU during the COVID-19 pandemic: an interview study and sentiment analysis. BMJ Qual Saf. (2021) 30:715–21. 10.1136/bmjqs-2020-011604 33028659 PMC8380894

[25] Hoffmann M Holl AK Burgsteiner H Eller P Pieber TR Amrein K. Prioritizing information topics for relatives of critically ill patients: cross-sectional survey among intensive care unit relatives and professionals. Wien Klin Wochenschr. (2018) 130:645–52. 10.1007/s00508-018-1377-1 30094664 PMC6244832

[26] Basch CH Fera J Pierce I Basch CE. Promoting mask use on TikTok: descriptive, cross-sectional study. JMIR Public Health Surveill. (2021) 7:e26392. 10.2196/26392 33523823 PMC7886372

[27] Southwick L Guntuku SC Klinger EV Seltzer E McCalpin HJ Merchant RM. Characterizing COVID-19 content posted to TikTok: public sentiment and response during the first phase of the COVID-19 pandemic. J Adolesc Health. (2021) 69:234–41. 10.1016/j.jadohealth.2021.05.010 34167883 PMC8217440

[28] Uhm JY Kim HS. Impact of the mother-nurse partnership programme on mother and infant outcomes in pediatric cardiac intensive care unit. Intensive Crit Care Nurs. (2019) 50:79–87. 10.1016/j.iccn.2018.03.006 29627395

